# Changes of aqueous humor cytokine profiles of patients with high intraocular pressure after PPV for retinal detachment

**DOI:** 10.1038/s41598-024-61913-7

**Published:** 2024-06-06

**Authors:** Chenchen Zhu, Yan Cheng, Yi Tang, Hong Wu, Zaoxia Liu

**Affiliations:** Eye Center of Second Hospital, Jilin University, 218 Ziqiang Street, Nanguan District, Changchun, 130041 China

**Keywords:** Retinal detachment, High IOP, Pars plana vitrectomy, Cytokines, PVR, Ocular hypertension, Retinal diseases, Vitreous detachment

## Abstract

High intraocular pressure (IOP) is one of the early complications after pars plana vitrectomy (PPV), which may cause glaucoma and poor visual prognosis secondary to surgery. Proliferative vitreoretinopathy (PVR) is one of the complications of retinal detachment (RD) and is the main reason for the poor prognosis, which is related to different kinds of cytokines. It’s essential for the basic mechanism to analyze the relative aqueous humor cytokine profiles with IOP after PPV for RD. In this study, we have collected the aqueous humor of 16 patients and qualified 27 cytokines using Luminex and compared biomarkers with the high IOP group and the normal group. As a result, the concentrations of VEGF, IL-6, FGF2, and G-CSF upregulated significantly (P < 0.05), while VEGFR2 downregulated significantly (P < 0.05) in the high IOP group. IL-6 was positively correlated with high IOP (r = 0.561, P = 0.041). Meanwhile, the concentrations of IL-6 (r = 0.543, P = 0.03), IL-5 (r = 0.576, P = 0.019), IL-15 (r = 0.614, P = 0.011), IL-4 (r = 0.517, P = 0.04), ICAM-1 (r = 0.611, P = 0.012), and G-CSF (r = 0.636, P = 0.008) were significantly associated with preoperative PVR classification, and the aqueous humor levels of IL-4 (r = 0.567, P = 0.022), HGF (r = 0.701, P = 0.005), and MCP-1 (r = 0.565, P = 0.035) are significant relative to laser points. Hence, cytokines might potentially be the therapeutic target of high IOP after PPV.

## Introduction

Pars plana vitrectomy (PPV) is a common surgical treatment of vitreoretinal disorders, and the surgical procedure includes peeling maneuvers, removing of tractions, and vitreous packing^1,2^. PPV is the most prominent modality for treating RD by removing vitreous fluid from the subretinal space, thereby restoring the adhesion of the neuroretinal epithelium to the pigment epithelium^3,4^. Transient or permanent IOP elevation is one of the early complications after PPV^5^. As reported, up to 50% of patients with macular fissures have developed high IOP after PPV^5^. Postoperative IOP elevation may cause glaucoma or poor visual prognosis secondary to surgery^6^. Although several studies have analyzed the aqueous humor cytokines profile of RD and the possible causes of high IOP after PPV surgery^7,8^, the mechanism of RD patients with high IOP after PPV is still unclear.

Photoreceptor cells, Müller cells, and immune cells heal the damaged retina through fibrosis and release cytokines to regulate RD-induced retinal damage. During RD, cytokines and chemokines participate in the pathophysiological processes^9^. Meanwhile, oxidative stress and inflammation are activated, and macrophages/microglia release cytokines and chemokines in active response to pathological changes in RD^10^. Researches have shown that MCP-1, TNF-α, and IL-1β are related to the development of inflammation and photoreceptor cell damage in RD^11–13^. RD causes vitreous fluid to flow into the subretinal space, an immune-privileged site maintained by the retinal pigment epithelium (RPE) and photoreceptors. When the subretinal space is severely damaged, photoreceptors undergo apoptosis and autophagy, while the RPE occurs in epithelial–mesenchymal transition (EMT)^14^ to promote tissue repair. Similarly, in the process of retinal healing and fibrosis, stressed RPE can recruit macrophages to release various inflammatory factors. At the same time, Müller cells produce growth factors such as VEGF, FGF2, and PEGF to promote wound healing^14^. The trabecular meshwork (TM) is a porous structure between the iris and cornea that regulates IOP by stretching the cell volume through mechanical stress to adjust aqueous humor outflow^15^. PPV triggers the oxygen levels increase in the vitreous cavity, which leads to oxidative stress damaging the TM^6^. Simultaneously, the repairing of damaged tissue promotes TM fibrosis and stiffness^15^. Stiffening of the trabecular meshwork increases mechanical stress, thereby increasing the release of cytokines and chemokines, including IL-1, IL-6, IL-8, TNF-α, MMP family, VEGF, etc., which regulate the outflow of aqueous humor and thus IOP^15^.

PVR is one of the complications of RD and is the main reason for the poor prognosis. PVR is a scarring process that accompanies retinal detachment and is the leading cause of the failure of PPV surgery^16^. Studies have shown that various factors such as MMP, N-cadherin, TNF-β, PDGF family, TNF, IL-6, and IFN-γ are involved in PVR and EMT processes^9^. In PVR, the blood-retinal barrier is disrupted, and RPE is exposed to growth factors and cytokines and thus loses polarity. The RPE then undergoes EMT to form the retinal adventitia^17^. Clinically relevant factors may be associated with increased postoperative IOP. Zhang et al. and Marti et al. have shown that silicone oil filling after PPV increases IOP, inducing glaucoma and even vision loss^18,19^. Intraoperative management also affects the prognosis of the procedure. Intraoperative condensation (cryotherapy) can damage retinal blood vessels, break down the blood-retinal barrier, promote the formation of anterior membranes, and cause retinal traction and even the risk of PVR^20,21^. Laser photocoagulation can enhance intraocular inflammation and stimulate intravitreal proliferation, exacerbating PVR^22^.

Luminex is a novel biomolecular assay technology that uses microspheres (beads) as the solid phase for molecular detection. By fluorescent staining of hundreds of microspheres and making fluorescence maps for detection, red fluorescence can be used to characterize cytokines and green fluorescence can be used to quantify cytokines^23^. The individually stained microspheres have monoclonal antibodies that target cytokines and chemokines and are detected by flow cytometry. Luminex has the advantages of high throughput, high sensitivity, small sample size and low cost for multi-sample analysis. Using Luminex for integrated monitoring and assessment of multiple cytokines can better reflect the overall changes in disease onset and progression. Luminex multiplex microsphere arrays are now being used to measure multiple aqueous humor factors simultaneously^24,25^.

Based on the above theory, in this prospective study, we collected the aqueous humor of RD patients with high IOP after PPV surgery and RD patients with normal IOP after PPV surgery. We proposed that aqueous humor factors are related to high IOP and clinical characteristics. Therefore, we selected the interleukin family associated with inflammation, cytokines associated with RD injury repair, EMT-related cytokines, and IOP-related cytokines for detection. We operated Luminex to detect cytokines and chemokines in the aqueous humor. Analyze the relationship between aqueous humor factors in patients with high IOP, PVR grading, laser points, and intraoperative condensation. To investigate the etiology and mechanism of postoperative hypertension and provide further research to improve surgical approach and prognosis.

## Methods

### Patients data

This investigation is a prospective study, including 16 cases of vitrectomy silicone oil filling for retinal detachment from September 2020 to November 2021. The experimental group involves 16 patients with high IOP after PPV surgery, and after the intraocular pressure returned to normal collect the aqueous humor as the control group.

Surgeries are performed under the surgical microscope, including 25G vitrectomy, laser photocoagulation, and silicone oil filling. Exclusion criteria: (1) History of intraocular surgery; (2) Patients already on anti-VEGF therapy; (3) History of trauma or other eye diseases; (4) Patients with retinitis and uveitis; (5) Family or personal history of glaucoma; (6) Patients with systemic diseases such as rheumatic disease or used steroid hormone.

Patient data included age (46.19 ± 13.41), gender (male:female, 14:2), preoperative IOP (13.94 ± 4.06 mmHg), retinal fissure location, lens status, refractive error (equivalent spherical lens), duration of subjective symptoms, duration of high IOP, presence, and grading of PVR. Among all of these samples, there were 3 patients with diabetes, 3 cases treated with insulin, and 2 patients with hypertension. All enrolled patients were first-time vitrectomies (Table 1).

**Table 1:** Baseline and clinical characteristic of study cohort.

Project	Value
Age (${\bar{x}}$ ± s)	46.19 ± 13.41
Gender (male/female)	14/2
Preoperative IOP (mmHg)	13.94 ± 4.06
Time from onset to operation (d)	35.87 ± 6.68
Time of first IOP after surgery (mmHg)	19 ± 2.09
Lens (yes/no)	16/0
Diabetes (yes/no)	3/13
Hypertension (yes/no)	2/14
Anti-VEGF infection (yes/no)	0/16
Iris neovascularization (yes/no)	0/16
Dialyze (yes/no)	0/16
History of eye surgery (yes/no)	0/16

The Ethics Committee of Jilin University approved the study and the study was in full compliance with the tenets of the Declaration of Helsinki, 1995 (as revised in Edinburgh, 2000). Each participant provided informed written consent for the use of their biological material and clinical data.

### Clinical characteristics

In this study, high IOP simultaneously satisfies IOP > 40 mmHg and is ineffective with IOP-reducing therapy. High IOP group: aqueous humor was obtained from patients with increased IOP after vitrectomy; control group: aqueous humor was obtained from high IOP patients whose IOP had returned to baseline after vitrectomy. For postoperative patients with high IOP, extracting aqueous humor with 1 mL insulin needles. Collect 0.05 mL aqueous humor through vitreous cavity puncture, and store in − 80 °C refrigerator. Meanwhile, record the patient's IOP and corresponding puncture time. For the control group, whose IOP is normal and the fundus is well, collect 0.05 mL aqueous humor per patient before silicone oil removal surgery. The other conductions are the same with high IOP group.

According to the “Retina Society Terminology Committee (1983)”^26^, we classified patients into 4 stages according to the severity of PVR: PVR A, PVR B, PVR C, and PVR D. In our study, we collected 6 patients with PVR grade B, 5 patients with PVR grade C, and 5 patients with PVR grade D. The samples of high IOP and control group are collected from the PVR patients.

### Luminex measure cytokines

The magnetic beads were coupled to the specific capture antibody according to the manufacturer’s (Merck Millipore, Germany) instructions and shaken overnight at 4 °C under light-proof conditions. The sample was incubated with streptavidin for 30 min, washed twice, and 100 μl of the mixture was added to the plate and analyzed by MagPlex instrument (Merck Millipore, Germany). We quantified 27 cytokines in each vitreous sample: IL-1β, IL-2, IL-4, IL-5, IL-6, IL-7, IL-8, IL-10, IL-12, IL-15, IL-17, VEGF, VEGFR2, PDGF-BB, IP-10, EGF, IFN-α, IFN-γ, CCL7, FGF, G-CSF, VCAM-1, ICAM-1, MCP-1, TNF-α, HGF, PIGF. On each test plate, we ran one replicate concentration standard for each cytokine in parallel.

### Statistics

All data were analyzed using IBM SPSS Statistics 22 (SPSS, IBM Corp, New York). We used the Shapiro–Wilk test to test the normal distribution of the data in each group, the Mann–Whitney test to compare the abnormal distribution data between the high IOP group and the control group, the *t*-test to compare the normal distribution data between the high IOP group and the control group, and the Spearman correlation analysis to analyze the relationship between aqueous humor factor and high IOP and the relationship between aqueous humor factor and laser points and condensation, using Pearson correlation analysis for data that conformed to a normal distribution and Spearman correlation analysis for data that did not conform to a normal distribution. p < 0.05 was considered to be significant.

### Ethics declarations

The Ethics Committee of Jilin University approved the study and the study was in full compliance with the tenets of the Declaration of Helsinki, 1995 (as revised in Edinburgh, 2000). Each participant provided informed written consent for the use of their biological material and clinical data.

### Informed consent

Informed consent was obtained from legal guardians of all individual participants included in the study.

## Results

### Baseline background

See Table 1.

### Comparison of cytokines profiles between high IOP and control group

**Figure 1:**
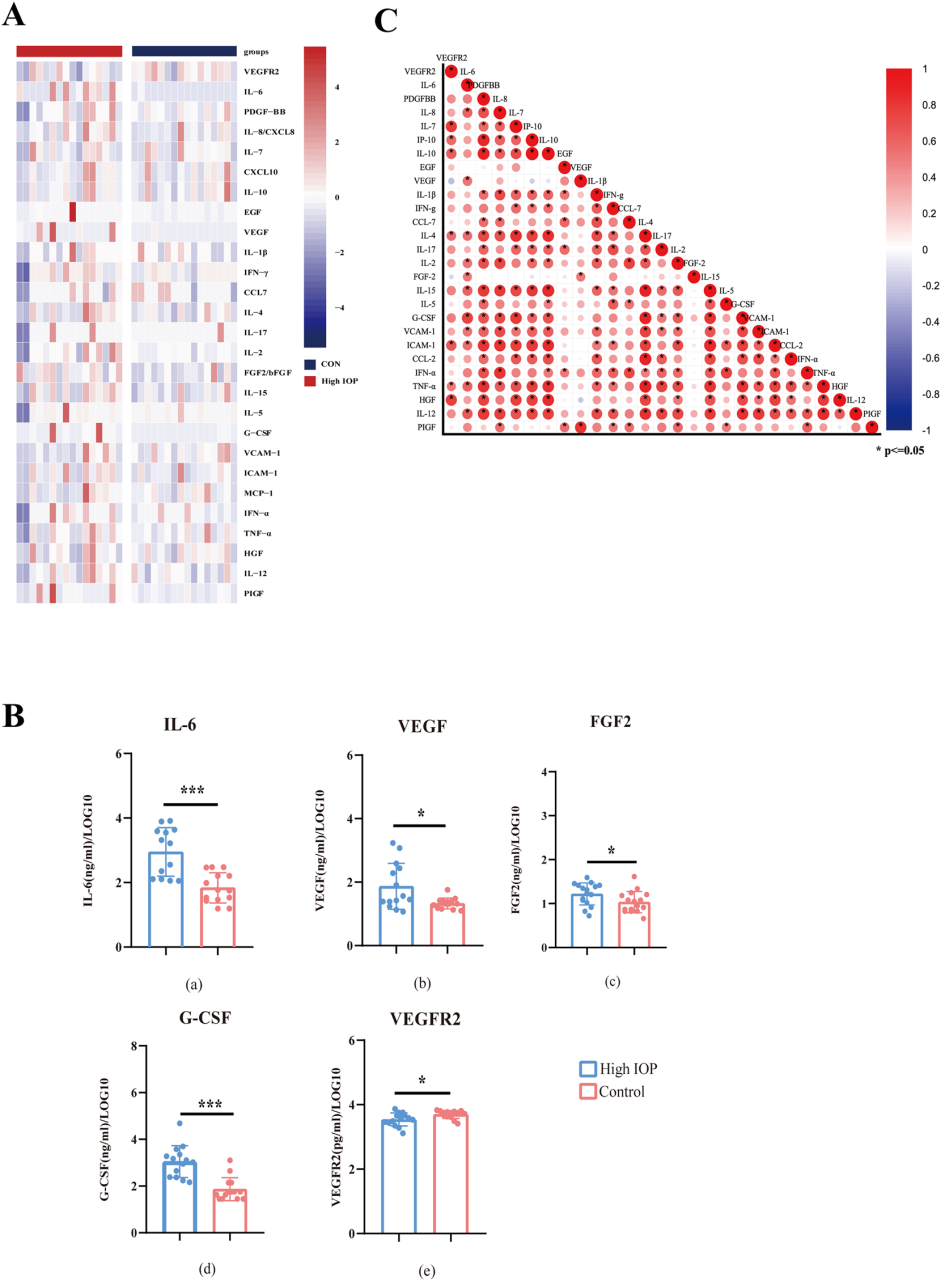
(**A**) Heatmaps for 27 cytokines measured in high IOP group and control group (CON). Red means up-regulation, blue means down-regulation. (**B**) Cytokines in the high IOP group and the control group. (**a**–**d**) Upregulated cytokines in high IOP. (**e**) VEGFR2 is lower in high IOP group than control group. (**C**) Relationship among cytokines in high IOP group. Red shows positively related and blue shows negatively related. *IL-6:* interleukin-6, *VEGF:* vascular endothelial growth factor, *FGF2:* basic fibroblast growth factor, *G-CSF:* granulocyte colony-stimulating factor, *VEGFR2:* vascular endothelial growth factor receptor 2; Use LOG10 to display the data. *P < 0.05, ***P < 0.001. Heatmap was conducted by R version 4.2.2 (2022-10-31 ucrt)—“Innocent and Trusting”. Copyright (C) 2022 The R Foundation for Statistical Computing. Figure (**C**) was conducted by Origin, Version 2022. OriginLab Corporation, Northampton, MA, USA.

In this study, we examined the profiles of 27 cytokines in 16 eyes in the high IOP and control group after RD surgery (Tables A.1 and A.2). Heatmaps display the discrepancy of each sample between high IOP and CON (Fig. 1A). VEGF (P = 0.027), IL-6 (P < 0.001), FGF2 (P = 0.033), and G-CSF (P < 0.001) were significantly upregulated in the postoperative high IOP group after RD (Mann–Whitney, P < 0.05), while VEGFR (*t*-test, P = 0.026) was significantly downregulated (Fig. 1B). IL-8, IL-15, and IL-4 slightly raised in the RD postoperative high IOP group (Fig. 1A), but there was no significant discrepancy compared with the control group. The other 19 cytokines were of no significance. Furthermore, analyze the relationship among cytokines in high IOP group (Fig. 1C). IL-6 is positively correlated with G-CSF, VEGF, and FGF-2 (P < 0.05). Meanwhile, in the growth factor family, VEGF was positively related to FGF2 (R = 0.505, P = 0.046). Describe the data using quartiles and means, respectively.

### Relationships between aqueous humor factors and clinical characteristic

**Figure 2:**
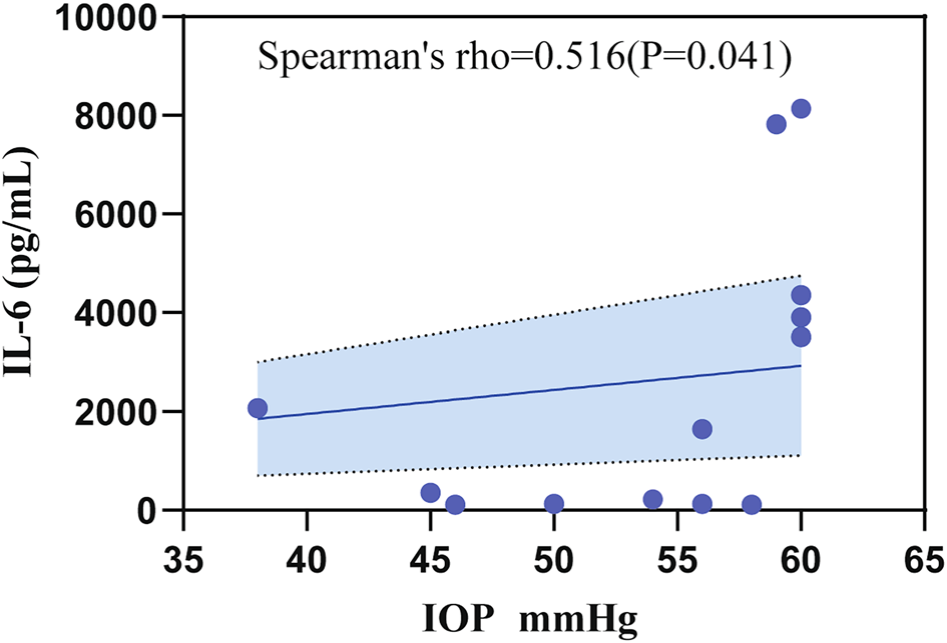
The relationship between IL-6 and high IOP. Spearman’s correlation. P < 0.05.

**Figure 3:**
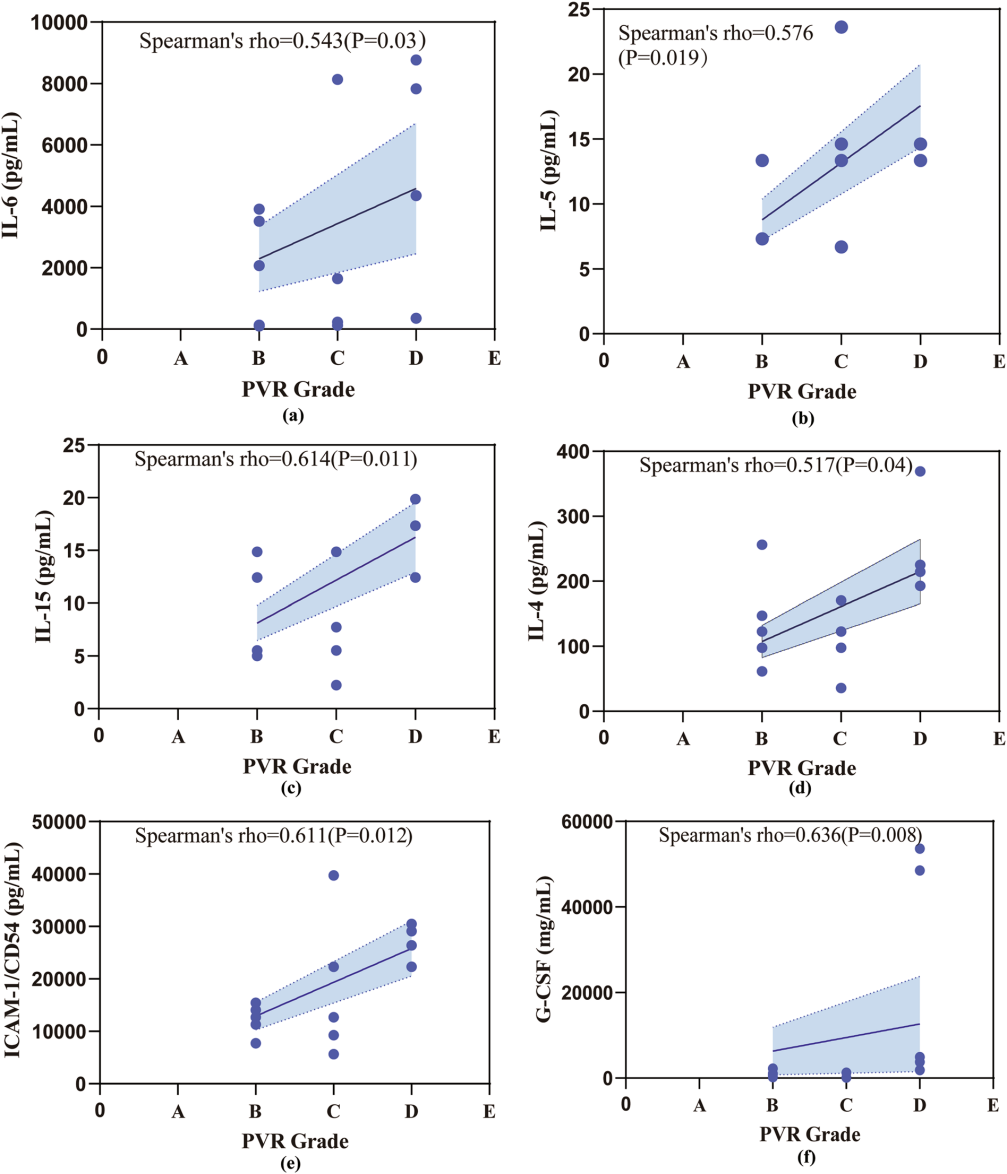
Relationship between cytokines in the high IOP group and PVR grading. *ICAM-1:* intracellular adhesion molecule, *G-CSF:* granulocyte colony-stimulating factor.

**Figure 4:**
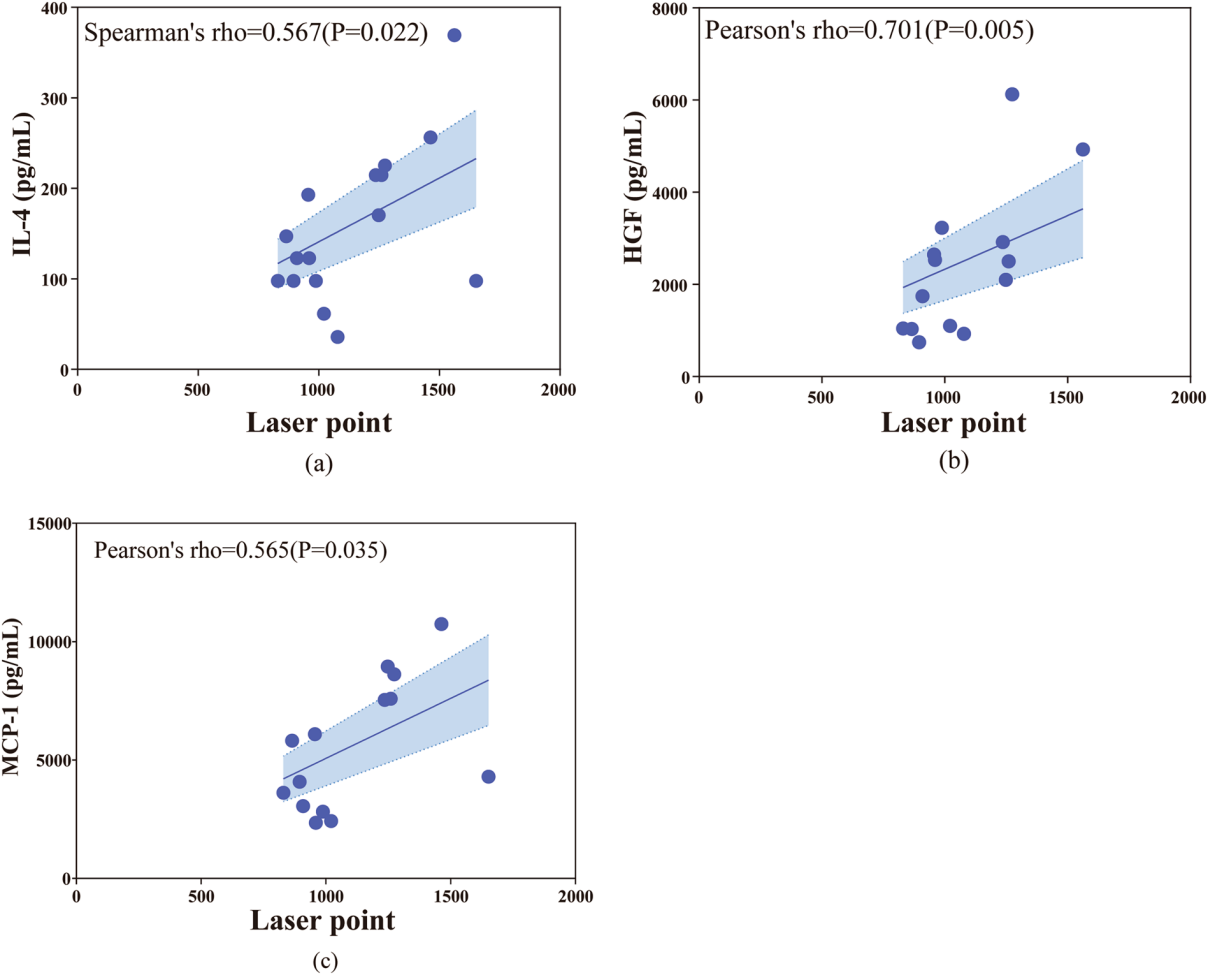
Relationship between cytokines in the high IOP group and laser points. *HGF:* hepatocyte growth factor, *MCP-1:* monocyte chemoattractant protein 1. P < 0.05.

In this study, we analyzed the relevance between cytokine profiles and clinical characteristics, including high IOP, laser points, and PVR classification in patients in the postoperative high IOP group after RD, and the results showed that IL-6 was significantly correlated with high IOP (Spearman's correlation coefficient = 0.516, P = 0.041) (Fig. 2). The aqueous humor level of IL-6, IL-4, IL-15, IL-5, G-CSF, and ICAM-1 had a significant positive correlation with preoperative PVR grading (Spearman’s correlation coefficient, P < 0.05) (Fig. 3). IL-4, HGF, and MCP-1 were significantly related to laser points (Spearman’s correlation coefficient, P < 0.05) (Fig. 4). However, the cytokine profile in the surgical condensation group showed an overall decreasing trend compared to the non-condensation-treated group, but there was no significant difference (Table A.3).

## Discussion

Our study analyzed the cytokine profile of aqueous humor in RD patients with high IOP after PPV surgery. The results showed that VEGF, IL-6, FGF-2, and G-CSF were significantly increased in RD patients with high IOP after PPV, while VEGFR2 significantly decreased in the high IOP group. In addition, we found that IL-6 has remarkable relation to high IOP. IL-6 signaling may affect IOP in mice by regulating the hydrodynamics of the aqueous humor or by influencing the development of the anterior chamber^27^. In the meantime, IL-6 was significantly associated with elevated VEGF, FGF-2, and G-CSF suggesting that IL-6 may be a key factor causing inflammation in RD patients with high IOP after PPV surgery. IL-6, IL-4, IL-15, IL-5, G-CSF, ICAM-1, and IFN-α were significantly correlated with the preoperative PVR grading of patients, and the higher the preoperative PVR grading, the higher the postoperative expression of IL-6, IL-4, IL-15, IL-5, G-CSF, ICAM-1, and IFN-α of patients showed an increasing trend.

The consequence supports IL-6 as a critical factor in RD and PVR pathology^17,28^. IL-6 responds to high IOP-promoted mechanical stress-induced retinal cell states^29^, while IL-6 may be a significant factor in RPE cell migration and fibrosis formation of the adventitia^17^. In this study, the level of IL-6 was the more discernible increase in the high IOP group than in the control group. With the increase of IOP, IL-6 increased significantly. Therefore, we reckon that IL-6 is pivotal in causing postoperative high IOP. IL-6 was significantly correlated with the severity of PVR. As the preoperative PVR grade grows, the concentration of IL-6 raises, which is in accordance with the findings of Qi et al*.*^17^, in which IL-6 as a pro-inflammatory factor promotes RPE cell migration and EMC, promoting tissue fibrosis to form PVR tissue. In our results, IL-6 correlated significantly with VEGF, FGF2, and G-CSF. In contrast, VEGF, FGF2, G-CSG did not connect with IOP and PVR grade, suggesting that IL-6, a key factor in inflammation and fibrosis, forms an inflammatory cytokine network with VEGF, FGF2, G-CSF and jointly regulates inflammation and repair postoperative PPV eyes.

The growth factors VEGF, FGF2, and G-CSF in the aqueous humor of the high IOP group were significantly increased compared with the control group, but the mechanism is still unclear. FGF2 is an essential cell fibro factor that stimulates the proliferation and migration of RPE and fibroblasts. In an earlier study, La Heij et al. compared aqueous humor factors in patients with net detachment and PVR, demonstrating that FGF2 is part of the mechanism of retinal damage^30^. G-CSF has a function of neuro-protective, which may have the potential to prevent glaucoma^25^. VEGF overexpression caused by vascular abnormalities may lead to neovascular glaucoma, which may cause ocular hypertension^31^. An evidence-based study has shown that anti-VEGF therapy can effectively reduce IOP^32^, and VEGF is closely associated with increased intraocular pressure. Combined with the results of this study, VEGF may be one of the reasons for the elevated IOP. VEGFR2 is a receptor of VEGF and a major mediator of mitosis and increased vascular permeability. In this study, VEGFR2 was significantly lower in the high IOP group than in the control group. The possible explanation is that VEGF signaling activation is mediated directly or indirectly by VEGFR and VEGFR-dependent crosstalk. In contrast, an extensive increase in VEGF inhibits VEGFR2 expression, while an increase in VEGFR1 expression inhibits VEGFR2 expression^33,34^. Wei et al*.*^35^ studied the release of VEGF, VEGFR1 and VEGFR2 in aqueous solution of patients after PPV, and the results showed that the expressions of VEGF and VEGFR1 increased significantly. At the same time, there was no significant difference in VEGFR2^35^. Therefore, we speculate that VEGFR2 may not be a target for anti-VEGF therapy in patients with intraocular hypertension after RD surgery. Our study shows that IOP may be the reason of cytokines. In the contrast, the increasing cytokines may influence the circling of humor aqueous. The relationship between growth factors and IOP after PPV surgery needs further exploration.

PVR is a complication of RD, meanwhile it’s a serious complication after surgery of RD, whose occurrence may be related to cryotherapy, laser, and PPV^36^. According to the data, PVR grading and laser count are positively related to the concentration of aqueous humor factors in ocular hypertension. In our findings, IL-4 are positively related to PVR stages and laser count. IL-4 is an anti-inflammatory factor increasing in primary open-angle glaucoma and neovascular glaucoma^37^. IL-4 can promote macrophages to transform into M2 type, promote tissue repair, and have anti-inflammatory effects^38^. As previously mentioned, fibrosis or EMT occurs during retinal repair, and fibrosis changes or extracellular matrix deposition can slow or prevent aqueous humor outflow, resulting in IOP rising. In addition to IL-4, the concentration of IL-6, IL-15, and IL-5 in the interleukin family increased with the severity of PVR. Additionally, in a study on biomarkers of PVR^39^, the concentration of IL-4 and IL-6 were increasing with the stage of PVR. ICAM-1 is expressed on fibroblasts and endothelial cells, involving intercellular and cell–matrix adhesion, and regulates cell migration processes^40^. It was demonstrated that high expression of ICAM-1 in the vitreous fluid is a high-risk factor in patients with RD who developed PVR^41^, consistent with our results in which ICAM-1 was positively correlated with the severity of PVR in RD patients with high postoperative IOP.

HGF is a hepatocyte growth factor that promotes RPE proliferation and migration^42^. And HGF promotes RPE proliferation in the subretinal fluid^43^. In studies of postoperative hypertension, HGF increased with an increasing number of laser points, suggesting that laser points cause hypertension in patients and increase the risk of glaucoma. MCP-1 is the primary mediator of photoreceptor apoptosis, a major cause of visual loss in several retinal disorders^13^. And Müller glia express MCP-1 increased in RD^13^, which is in accordance with our results that MCP-1 is related to laser points in RD patients.

In conclusion, VEGF, IL-6, FGF2, and G-CSF were elevated in the postoperative high IOP group and statistically different from the control group. The inflammatory factors (IL-6, G-CSF) and growth factors (VEGF, VEGFR2, FGF2) might be dominant in postoperative high IOP. Furthermore, our findings revealed a significant association between IL-6 and high IOP, indicating its crucial role in the inflammatory network of high IOP. Analysis of changes in aqueous humor factor levels related to PVR classification, condensation, and laser points contributed to inferring the degree of damage repair to photoreceptor cells and retina following surgery, thus contributing to the screening of potential therapeutic targets for improved postoperative prognosis. However, the study has a limitation of sample size and disease groups. Meanwhile, further experiments to verify the relationship between cytokines and IOP are necessary. The changes in hemodynamics after PPV are important in the prognosis of the eye^2,44^, and the relationship between the changes in blood vessel density and cytokines is worth further investigation. Expanding the disease group and sample size may make more discoveries in biomarkers and clinical factors.

### Supplementary Information

Supplementary Information.

Supplementary Tables.

## Data Availability

All data generated or analyzed during this study are included in this article (and its Supplementary Information files).
