# Supplementary material for: Changes of aqueous humor cytokine profiles of patients with high intraocular pressure after PPV for retinal detachment
Source: Sci Rep. 2024 Jun 6;14:13044. doi: 10.1038/s41598-024-61913-7 (PMC11156964; doi:10.1038/s41598-024-61913-7)
Supplement: Supplementary file 2 — Supplementary Tables. [file 41598_2024_61913_MOESM2_ESM.doc]

Supplementary information

**Table A.1.** Aqueous humor level of Cytokines in the high IOP group and the control group.

| **Cytokines** | **50% (25%, 75%)** | | | **P value** |
| --- | --- | --- | --- | --- |
|  | **High IOP** | | **Control** |  |
| IL-6 | 176.175(117.6675，1609.3125) | | 34.26(21.27，58.085) | <0.001* |
| PDGF-BB | 9.42(7.9075，10.04) | | 9.42(8.8，10.04) | 0.969 |
| IL-8/CXCL8 | 186.14(71.365，305.1225) | | 89.14(73.61，137.79) | 0.305 |
| CXCL10/IP-10 | 425.14(230.01，785.3225) | | 472.51(244.59，805.95) | 0.89 |
| EGF | 5.05(5.05，7.495) | | 5.22(4.88，6.27) | 0.662 |
| VEGF | 144.895(13.025，955.4475) | | 24.5(14.755，28.405) | 0.027* |
| IL-1β | 11.81(9.0425，11.81) | | 11.81(8.12，11.81) | 0.855 |
| IFN-γ | 41.46(35.4975，41.46) | | 41.46(37.485，41.46) | 0.537 |
| IL-2 | 35.185(28.5025，44.4175) | | 37.93(32.44，37.93) | 0.259 |
| FGF2/bFGF | 23.275(9.085，29.14) | | 11.22(7.035，15.625) | 0.033* |
| IL-15 | 12.43(8.905，14.26) | | 7.73(5.53，11.235) | 0.36 |
| IL-5 | 13.35(13.35，14.295) | | 13.35(13.35，14.61) | 0.849 |
| G-CSF | 974.83(404.985，1375.25) | | 42.35(28.27，57.36) | <0.001* |
| IFN-α | 5.13(4.83，5.13) | | 5.13(4.73，5.96) | 0.381 |
| TNF-α | 8.72(8.02，10.5225) | | 9.42(9.42，10.89) | 0.789 |
| PIGF | | 6.235(4.78，19.1125) | 5.33(4.79，6.94) | 0.382 |

Mann-Whitney test. The results are expressed in quartiles. *P < 0.05 was considered statistically significant.

**Table A.2**. Aqueous humor level of Cytokines in the high IOP group and the control group.

| **Cytokines** | **Mean ± standard deviation** | | **P value** |
| --- | --- | --- | --- |
|  | **High IOP** | **Control** |  |
| VEGFR | 3799.0714±1660.07013 | 5131.9286±1248.88144 | 0.024* |
| IL-7 | 16.6575±7.80674 | 17.09±6.53654 | 0.866 |
| IL-10 | 42.82±26.32946 | 42.84±27.12516 | 0.998 |
| IL-4 | 157.7594±84.82425 | 131.1379±42.74489 | 0.298 |
| VCAM-1 | 18251.0714±7692.21055 | 15133.6429±5115.11162 | 0.218 |
| ICAM-1 | 132607.7857±105569.16382 | 175009.5714±99732.70319 | 0.285 |
| MCP-1 | 5571.2143±2739.51333 | 5853.9286±2111.25276 | 0.762 |
| HGF | 2397.205±1572.08853 | 2438.7329±992.97864 | 0.934 |
| IL-12 | 1186.0914±357.21999 | 1059.49±286.03193 | 0.290 |

Independent samples t-test. Results are expressed as mean difference ± SD. *P < 0.05 was considered statistically significant.

**Table A.3**. Mean ± standard deviation of cytokine profiles in the RD surgical condensation and non-condensation groups in the high IOP group.

| **Cytokines** | **Mean ± standard deviation** | | **P value** |
| --- | --- | --- | --- |
|  | **non-condensation** | **condensation** |  |
| VEGFR2 | 3977.5455±556.38857 | 3144.6667±151.76773 | >0.05 |
| IL-6 | 3147.3078±1086.08681 | 1046.6925±479.19835 | >0.05 |
| PDGF-BB | 10.1582±1.08289 | 9.306±0.48727 | >0.05 |
| IL-8/CXCL8 | 257.0644±50.40647 | 151.498±56.17102 | >0.05 |
| IL-7 | 16.1582±1.86856 | 17.756±5.10557 | >0.05 |
| CXCL10 | 742.8233±206.12345 | 466.866±115.62005 | >0.05 |
| IL-10/IP-10 | 46.7±9.79213 | 35.836±9.2978 | >0.05 |
| EGF | 10.6582±5.44602 | 5.536±0.26626 | >0.05 |
| VEGF | 330.997±191.151 | 175.12±92.09519 | >0.05 |
| IL-1β | 12.4055±1.56421 | 10.334±0.90386 | >0.05 |
| IFN-γ | 37.1482±3.93938 | 43.14±1.68 | >0.05 |
| CCL7 | 81.2991±8.50912 | 82.21±0 | >0.05 |
| IL-4 | 158.3409±29.04993 | 156.48±27.46505 | >0.05 |
| IL-17 | 11.7691±1.02614 | 13.604±1.314 | >0.05 |
| IL-2 | 38.0155±4.56827 | 35.422±4.00001 | >0.05 |
| FGF2 | 17.99±3.00421 | 21.588±4.13894 | >0.05 |
| IL-15 | 11.5936±1.84597 | 12.582±2.73079 | >0.05 |
| IL-5 | 13.3573±1.31294 | 14.358±0.252 | >0.05 |
| G-CSF | 1735.6678±552.07833 | 10350.534±9553.63946 | >0.05 |
| VCAM-1 | 159431.3333±40837.43951 | 84325.4±18272.13014 | >0.05 |
| ICAM-1 | 18831.8889±2605.03165 | 17205.6±3688.52596 | >0.05 |
| CCL2/ MCP-1 | 5339.4444±1042.26919 | 5988.4±948.55293 | >0.05 |
| IFN-α | 5.1891±0.44817 | 5.74±0.71421 | >0.05 |
| TNF-α | 10.2827±1.13739 | 10.662±1.1841 | >0.05 |
| HGF | 2286.187±400.11855 | 2674.75±1200.31589 | >0.05 |
| IL-12 | 1220.6611±117.32006 | 1123.866±178.5716 | >0.05 |
| PIGF | 8.0618±2.14803 | 11.97±5.99268 | >0.05 |
